# Bidirectional wavelength-division multiplexing transmission over installed fibre using a simplified optical coherent access transceiver

**DOI:** 10.1038/s41467-017-00875-z

**Published:** 2017-10-19

**Authors:** M. S. Erkılınç, D. Lavery, K. Shi, B. C. Thomsen, R. I. Killey, S. J. Savory, P. Bayvel

**Affiliations:** 1 Optical Networks Group, Dept. of Electronic and Electrical Eng., UCL, London, WC1E 7JE UK; 2 University of Cambridge, Dept. of Eng., Electrical Eng. Division, 9 JJ Thomson Avenue, Cambridge, CB3 0FA UK

## Abstract

High-speed broadband services require optical fibres in access networks, in which multiple subscribers are connected to service providers, to satisfy the continuously growing bandwidth demand. The primitive signaling scheme used in access networks enables the use of low-cost equipment but diminishes the bandwidth available to end-users. Thus, current technology will be unable to support future broadband demands. Coherent communication systems offer significantly improved power- and bandwidth-efficiency, but require fundamental simplifications to become economically viable for access networks. Here, we demonstrate a promising simplified coherent receiver exhibiting a robust performance against polarisation fluctuations over an installed fibre network. It enables the realisation of high-order modulation formats and offers high sensitivities, achieving a four-fold increase in the supported number of subscribers and approximately doubling the transmission distance compared to the recently standardized access technology. The proposed solution indicates that digital coherent technology can be feasible and transform the access networks, enabling ubiquitous new services and applications with uncontended, multi-gigabits/user broadband connections.

## Abstract (web summary)

The signalling scheme used in access networks require electrical bandwidths many times greater than subscribers can utilise. Here, the authors describe a promising approach to achieve bidirectional transmission with bandwidth-efficient yet low-complexity coherent optical network unit transceiver.

## Introduction

**Fig. 1:**
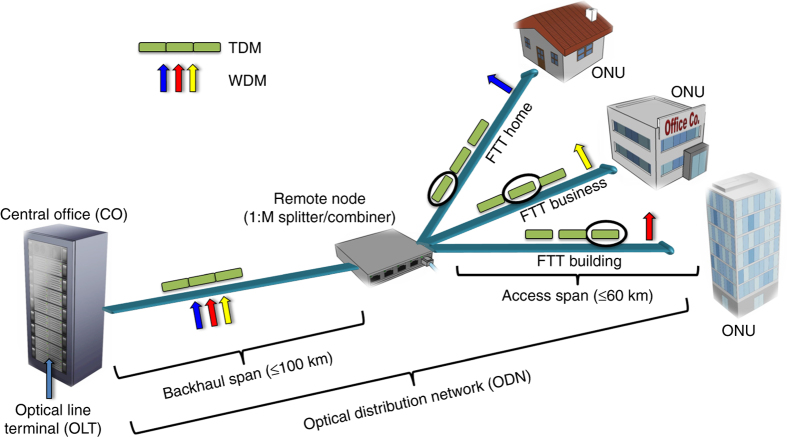
Schematic of a typical passive optical access network. Optical line terminal (OLT), installed by a service provider, distributes a TDM or WDM signal via ODN, consisting of transmission fibre and passive splitters/combiners. ODN consolidates the backhaul and access spans which are typically up to 100 km. The optical network units located at business/building/premise, or home receive the transmitted signal and provide bandwidth to each fixed (e.g., personal computers, and IP-TVs) and mobile (e.g., smartphones, tablets, and laptops) end user supported by the network. ONU: optical network unit, FTTx: Fibre-to-the-Business/Building/Premise/Home

Network operators continue to seek solutions to increase the capacity of optical access networks to meet the ever-increasing bandwidth demands, driven by bandwidth-hungry applications, such as high-definition video-on-demand, entertainment (e.g., online gaming) and Internet of Things. Fibre-to-the-Home/Premise/Business or Building (FTTx) is widely viewed as the only access network technology capable of meeting this demand. However, it is highly cost-sensitive since the cost of an overall access network is born solely by the end users supported in the network. Therefore, the most attractive optical access network architecture is the passive optical network (PON) with a tree topology, in which the overall complexity is minimised by employing only passive splitters/combiners in a remote node to provide broadband access to the subscribers, i.e., using no active components, such as electronic switches/routers. In addition, the transmission fibre can be utilised bidirectionally, i.e., the downstream and upstream signals counter-propagate in the same fibre. Such passive optical access networks have been widely deployed since 2004^1^. Most of the PON solutions have a point-to-multi-point architecture and connect a service provider's central office (consisting of several optical line terminals) to multiple subscribers, using so-called optical network units (ONUs), via an optical distribution network, as depicted in Fig. 1.

Current PON FTTx technologies such as 10 Gigabit-PON, 10 G Ethernet-PON utilise time-division multiplexing (TDM), and the recently announced next generation PON technology (NG-PON2), offering at least 40 Gb/s (4*λ* × 10 Gb/s) aggregate network capacity^2–5^, will employ time-wavelength division multiplexing^6, 7^ coupled with direct detection receivers. However, TDM transceivers used in ONUs operate at aggregate data rates rather than the data rate per subscriber. Hence, they require electrical bandwidths many times greater than the bandwidth that subscribers can utilise^6^, e.g., providing just 150 Mb/s per user (in a network supporting 64 users) by using a receiver requiring an electrical bandwidth of 10 GHz (≈150 Mb/s × 64 users). Such signal orthogonalisation schemes will be inevitably limited when the demand for bandwidth reaches uncontended multi-gigabit/s per subscriber. To avoid bandwidth exhaust in PONs, the utilisation of the wavelength-to-the-user approach, i.e., dedicating a single wavelength to each user as illustrated in Fig. 1, will be the ultimate solution for the next generation PONs, referred to as wavelength-division multiplexing (WDM) PONs^8–11^.

In PONs, the received signal power is relatively low, and hence, achieving high receiver sensitivity is a key challenge. However, direct detection systems are limited by receiver noise (mainly thermal noise, but also limited photodiode responsivity/gain). Thus, the advanced direct detection schemes offering higher bandwidth efficiency will exhibit a lower receiver sensitivity in unamplified systems vs., for example, on-off keying (OOK). Fortunately, this limitation can be overcome through the use of coherent receivers. Besides the power efficiency, coherent technology inherently enables ONUs with wavelength/frequency selectivity, e.g., offering 10 Gb/s per user by employing a coherent receiver with a bandwidth of ≤10 GHz depending on the achievable spectral efficiency. Moreover, it offers significant advantages compared to the currently employed intensity modulation/direct detection transceivers such as linear optical field detection, high achievable spectral efficiency, and robustness to chromatic dispersion^12^.

Future PONs will require the availability of high-performance, cost-effective coherent receivers which can be shown to be feasible in real fibre networks, exhibiting polarisation and temperature fluctuations; to date, this has not been achieved. The optical complexity of conventional polarisation- and phase-diverse intradyne coherent receiver employed in core and long-haul networks is considered to be excessively high due to the use of polarisation beam splitter(s)/rotator(s) and 90° optical hybrids. Therefore, the complexity is the main limiting factor that prevents its use in ONU transceivers for PON applications. Although the single-chip monolithic integration of a conventional coherent receiver is possible^13, 14^, it is challenging using mature manufacturing techniques for volume production. Thus, low-complexity monolithically integrable coherent receivers maintaining high receiver sensitivity can potentially be the fundamental driving force of future coherent-enabled PON systems. Recently, Cano et al.^15^ demonstrated a coherent-enabled PON operating at 1.25 Gb/s effective user bit rate using a simplified (heterodyne) coherent receiver over a transmission distance of 50 km, and tested it in a field trial (10 km)^16^. Following this, the per-user connection speed was increased to 10 Gb/s employing the same receiver architecture and transmitted over 25 km^17^. However, such demonstrations do not exhibit a bidirectional transmission, implying that a second laser in an ONU might be required for the upstream signal generation. On the other hand, we have proposed and presented the principle of operation of a single-polarisation balanced coherent receiver, requiring minimum optical complexity^18^. It avoids power fading due to the polarisation rotation, enabled by performing polarisation-time block coding coupled with heterodyne detection. The preliminary performance evaluation of the proposed receiver was assessed in a bidirectional WDM-PON system^19^.

The main contribution of this work is the installed fibre network demonstration of bidirectional dense WDM transmission using quadrature phase-shift keying (QPSK) and 16-quadrature amplitude modulation (QAM). This is realized by the full implementation of a bandwidth-efficient yet low-complexity coherent ONU transceiver, implemented through the use of a polarisation-time block coding using digital signal processing (DSP) combined with heterodyne detection. It is shown that the proposed receiver exhibits robust performance in the presence of fluctuations in the polarisation state of the received signal, as typically occurs in installed fibres, thereby offering high receiver sensitivities and enabling the realisation of bandwidth-efficient modulation formats. The proposed transceiver has a 75% lower optical complexity, quantified by the number of balanced detectors, compared to conventional coherent receivers, which potentially eases the monolithic integration whilst preserves the benefits of coherent technology, e.g., exceptionally high receiver sensitivity, robustness to chromatic dispersion, and colourless receiver operation due to its frequency/wavelength selectivity. Some complexity is moved from the optical to the digital domain whilst maintaining the linearity between the domains since the cost of silicon-CMOS technology continues to decrease, with increasing performance (according to Moore’s law). The photonic integration however, does not have the potential to scale comparably with the CMOS technology. Thus, it is likely that high-bandwidth DSP will be deployed even for low-cost short and medium reach applications in the near future. Moreover, it offers significantly higher loss budget and a comparable optical complexity to the currently employed ONU transceivers, as outlined in the Discussion section. In particular, we demonstrate a bidirectional dense WDM-PON transmission over a re-configurable installed dark fibre network, operating at a net aggregate data rate of 160 Gb/s (8 × 20 Gb/s) downstream and 80 Gb/s (8 × 10 Gb/s) upstream over a 37.6 km installed fibre network (compatible with future PON technologies). This demonstration is a proof-of-principle for 400 GbE PON, only requiring another 12 wavelengths which can be comfortably accommodated with the achieved loss budget, offering a four-fold increase in the number of subscribers or a doubling in the transmission distance compared to the next-generation PON technology.

## Results

### Principle of operation and Alamouti coding

**Fig. 2:**
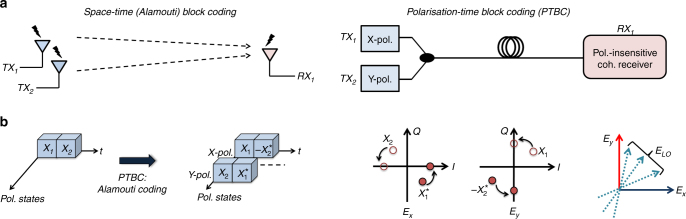
Realisation of Alamouti coding. **a** The analogy between wireless (two transmit antennae) and optical fibre communication (dual-polarisation transmitter), corresponding to a multiple-input-single-output system. **b** Alamouti polarisation-time block coding (PTBC) scheme and the illustration of polarisation rotation. *E*_*X*_ and *E*_*Y*_ correspond to the optical fields carrying the information and coded symbols ($\left[ {{X_1}\quad {X_2}} \right]$ and $\left[ { - X_2^*\quad X_1^*} \right]$) in X- and Y-polarisation modes, generated using transmitter 1 and 2 (*TX*_1,2_). Subsequently, the transmitted signal beats with the local oscillator *E*_LO_ in the receiver (*RX*_1_). *I* in-phase, *Q* quadrature

Alamouti coding is a space-time block coding scheme, and was first proposed to achieve two-branch transmit diversity using a single receiver in wireless communications^20^. It has been adapted for optical fibre communication systems using a conventional coherent intradyne receiver^21^ by drawing an analogy between the two transmit antennae and the two polarisation modes, as depicted in Fig. 2a. Note that such a design can be seen as a multiple-input-single-output system. The key idea is to divide a sequence of symbols into pairs over two polarisation, e.g., X- and Y-polarisation, modes, referred to as polarisation-time block coding, and to send the same information twice during two symbol duration, as illustrated in Fig. 2b. The symbol pairs are coded such that the information symbols are transmitted over two polarisation modes [*E*_*X*_ and *E*_*Y*_] as $\left[ {{X_1}\quad {X_2}} \right]$, $\left[ {{X_3}\quad {X_4}} \right]$ whilst their reciprocal pairs ($\left[ { - X_2^*\quad X_1^*} \right],\left[ { - X_4^*\quad X_3^*} \right]$) are sent in the next time slot where ^*^ represents the complex conjugate. Note that the symbol pairs transmitted over X- and Y-polarisation modes are mutually orthogonal to those transmitted in the next time duration. The detailed comprehensive ﻿discussion regarding the Alamouti coding scheme can be found in our paper^22^. Although this coding scheme introduces 50% redundancy due to the replication of the transmitted symbols and requires a polarisation modulator in the transmitter, it does not require any additional DSP complexity, higher bandwidth or resolution of the DACs/ADCs. Furthermore, it enables to remove the polarisation beam splitter(s)/rotator(s), the 90° optical hybrid and two of the balanced photodetectors which are used in a conventional polarisation- and phase-diverse coherent receiver. This approach can be attractive in applications such as access networks, in which asymmetrical transceiver architectures are typically employed (i.e., higher complexity transceivers can be employed in the Central Office than the ONUs). Furthermore, the polarisation-independent single polarisation coherent receiver enabled by Alamouti coding reintains many of the advantages of coherent detection including high power/bandwidth efficiency, linear optical field detection, and robustness to fibre impairments.

The polarisation of the optical field undergoes polarisation rotation along the fibre link during transmission, as illustrated in Fig. 2b. Since the beating between the transmitted signal [*E*_*X*_ *E*_*Y*_] and the local oscillator (LO) laser *E*_LO_ is the superposition of the polarisation modes from both transmitters, the information symbols (*X*_1_, *X*_2_, *X*_3_, *X*_4_, …) can be successfully recovered, independently from the state of polarisation of the received signal and *E*_LO_^22^. Consequently, this feature makes the detection process independent of the polarisation rotation which occurs during transmission. The coding scheme therefore removes the need for any optical polarisation tracking unit in the receiver such as a PBS, polarisation rotator or controller. This leads to a significant reduction in optical complexity compared to the conventional polarisation- and phase-diverse coherent receiver architecture, allowing the implementation of the proposed simplified coherent receiver using the minimum number of optical components possible, as further described in the Discussion section.

### ODN/OLT and ONU transceiver implementations

**Fig. 3:**
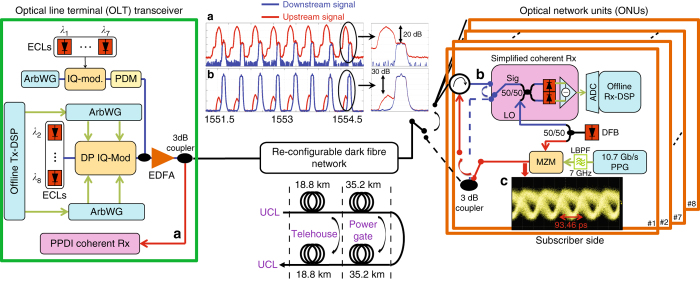
Bidirectional 8-channel 50 GHz-spaced dense WDM-PON transmission test-bed. The downstream channels were modulated with a 10.7 Gb/s Alamouti-coded OFDM QPSK and 16-QAM, whereas 10.7 Gb/s BPSK signal whose eye diagram was shown in the inset **c** was used for upstream channels. The frequency offset between the downstream and upstream channels was chosen to be 12.5 GHz, as shown in the insets **a**, **b**. The network was configured to first 37.6 (from UCL to Telehouse and back to UCL) and then 108 km (from UCL to Power gate via Telehouse and back to UCL via the same route). When the transmission distance was set to 108 km, the control switch in the ONU was changed from the 3-dB coupler to the optical circulator. It is worth noting that particularly in the ONU, a 3-dB coupler, which has 20–30 dB less isolation compared to an optical circulator, is more preferable. *Blue lines* indicate the downstream signal, whereas the upstream optical signal is represented using *red lines*. Insets: The received optical spectra in the **a** ONU and **b** OLT at 108 km. **c** Eye diagram of 10.7 Gb/s BPSK signal. LPF: low-pass filter, PDM: polarisation-division multiplexing, PPDI Rx: polarisation-and phase-diverse intradyne coherent receiver, UCL: University College London, VOA: variable optical attenuator

The schematic of the bidirectional dense WDM-PON transmission test-bed is depicted in Fig. 3. In the proposed configuration, eight downstream (DS) and upstream (US) channels were placed on a 50 GHz frequency grid with a 12.5 GHz offset, i.e., ${\lambda _{{\rm{D}}{{\rm{S}}_1}}}{\rm{/}}{\lambda _{{\rm{U}}{{\rm{S}}_1}}}$ = 1551.2/1551.1 nm and ${\lambda _{{\rm{D}}{{\rm{S}}_8}}}{\rm{/}}{\lambda _{{\rm{U}}{{\rm{S}}_8}}}$ = 1554.0/1553.9 nm, as shown in the insets (a) and (b) in Fig. 3. It is crucial to note that the frequency offset, which is used to avoid significant penalties due to the back-reflections in the ONU, was enabled by heterodyne detection and chosen such that it allows the simultaneous use of the ONU laser as a LO laser for the DS and the source laser for the US channel, as shown in Fig. 3. The optimisation of frequency offset between the DS and US channels is further discussed in the Back-to-back performance evaluation section.

The optical distribution network (ODN), emulating the back-haul and access spans, was used to transmit the DS and US signals over passive sections of a re-configurable dark fibre network, installed between University College London, Telehouse and Powergate, as shown in Fig. 3. Two demonstrations were performed to evaluate the system performance. In the first transmission experiment, the network was configured to have a typical PON reach (37.6 km of standard single mode fibre (SSMF) with 10 dB link attenuation); 8 DS channels (first Alamouti-coded orthogonal frequency-division multiplexing (OFDM) with QPSK, and then switched to 16-QAM subcarriers, operating at 10.7 and 21.4 Gb/s, respectively) and 8 US channels (binary phase-shift keying (BPSK) operating at 10.7 Gb/s) were bidirectionally transmitted over the installed fibre. Following this, a bidirectional 10.7 Gb/s symmetric long-reach dense WDM-PON transmission experiment was carried out by changing the network configuration to 108 km of SSMF with a total link attenuation of 27.6 dB. The link attenuation and average chromatic dispersion coefficients of the installed fibre were 0.26 dB/km and 16.5 ps/nm/km at 1550 nm, respectively.

The optical line terminal (OLT) transceiver implementation is as follows: The OLT transmitter for the even channels (*λ*_2,4,6,8_) consisted of a dual-polarisation nested Mach-Zehnder (IQ) modulator, seeded with the output from external cavity lasers with a linewidth of 100 kHz. Note that it is acceptable to use a narrow linewidth laser in the OLT since the cost is shared by the users supported in the network. Nevertheless, the linewidth tolerance of the proposed simplified coherent receiver has been investigated up to 10 MHz^23^ in which additional 0.5 and 1.5 dB reductions in the receiver sensitivity were observed at a linewidth of 2 MHz for Alamouti-coded OFDM QPSK and 16-QAM signals, respectively. It is worth noting that the combined linewidth of the system can be assumed to be ~2 MHz if a distributed feedback (DFB) laser with a linewidth of 1 MHz is used, the same as the ONU laser. The modulator was driven using an Alamouti-coded OFDM signal with subcarriers modulated, initially with QPSK, and subsequently, 16-QAM. The electrical driving signals were generated using an arbitrary waveform generator, operating at 12 GSa/s with an effective number of bits (ENOB) of 5-bit at 4 GHz. The signal waveforms were generated offline and the required DSP is explained in the Methods section. The odd channels were generated in a similar manner using a single-polarisation IQ-modulator, followed by the polarisation multiplexing delay-line emulation stage (decorrelating the second polarisation by 120 symbols). Subsequently, odd and even channels were coupled to form an 8-channel 10.7 and 21.4 Gb/s Alamouti-coded OFDM QPSK and 16-QAM signals, respectively, assuming a 7% overhead due to hard decision forward error correction (HD-FEC), as shown in Fig. 3. An Erbium-doped fibre amplifier with a noise figure of 5 dB followed by a variable optical attenuator were used to control the launch power of the DS channels into the fibre.

In the OLT receiver side, the US (8 × 10.7 Gb/s BPSK) signal was detected using a polarisation- and phase-diverse intradyne coherent receiver. The received signal was digitised using an analogue-to-digital converter (ADC) with a 3-dB electrical bandwidth of 20 GHz (operating at a sampling rate of 50 GSa/s), followed by a 7 GHz LPF. The required sampling rate for the received US signal was 21.4 GSa/s. The DSP was performed offline, and is detailed in the Methods section.

In the ONU transceiver, the DS signal was detected using the simplified coherent receiver, consisting of a 3-dB coupler and a single balanced photodiode, implemented using discrete optical components. The DFB laser with 1 MHz linewidth was split using a 3-dB splitter. One of its output ports was coupled with the received DS signal operating as a LO laser at a power of 10.5 dBm, as shown in Fig. 3. Following this, the signal was digitised using a single ADC, operating at 50 GSa/s with 3-dB electrical bandwidth of 20 GHz and an ENOB of 5-bit at 10 GHz. The required sampling rate for the DS channels was 40 GSa/s due to the simultaneous use of the ONU (both for DS LO and US transmitter) laser enabled by heterodyne detection. The offline DSP for the demodulation of the received DS signals (Alamouti-coded OFDM QPSK and 16-QAM) is described in the Methods section.

The ONU transmitter utilised the second output port of the 3-dB split DFB laser to generate the US signal, as shown in Fig. 3. The output was used as a source laser for the single-drive MZM with a 3-dB bandwidth of 10 GHz which was driven by a pulse pattern generator operating at 10.7 Gb/s. The modulator was biased at its null point to generate the BPSK signal, as shown in the inset (c) of Fig. 3. The bandwidth of the US transmitter electronics was limited to 7 GHz using a Bessel low-pass filter (LPF) to densely place the US and DS signals, as discussed above. It is crucial to note that a conventional 3-dB coupler with a 30 dB return loss was sufficient to combine the DS and US signals over 37.6 km transmission in the ONU, thanks to the achieved high receiver sensitivity enabled by the simplified coherent receiver. It was replaced with an optical circulator when the link length was increased to 108 km. This issue is further discussed in the Transmission demonstrations section.

### Sensitivity evaluation in back-to-back operation

**Fig. 4:**
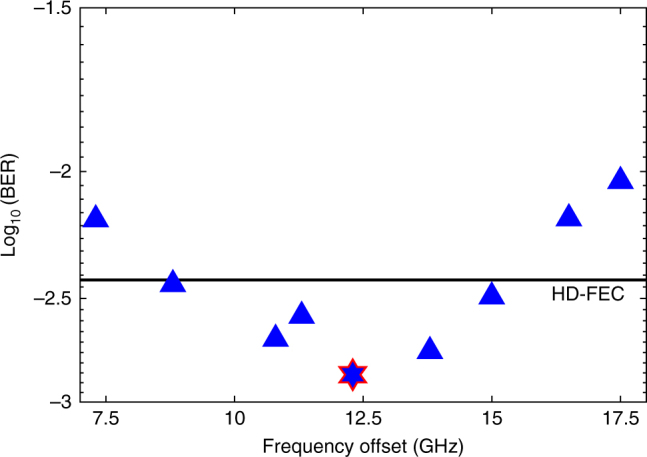
Achieved BERs with respect to the frequency offset. The performance of the DS signal at −40 dBm received power in single-channel operation vs. the frequency offset between the DS and US channels, obtained by varying the ONU laser wavelength

First, the optimum frequency offset between the DS and US channel was determined in single channel bidirectional operation by monitoring the bit error ratio (BER) performance of the DS channel in the presence of the US signal. It was tested with US transmitter powers of up to 15 dBm using the method described in ref. ^24^. The frequency offset was controlled by varying the ONU laser wavelength, operating at 10.5 dBm power at the photodiode input. It should be noted that such high LO power managed to be launched since the insertion loss of the proposed simplified coherent receiver, requiring only a 3-dB coupler (no PBSs and 90° hybrids), is ~7 dB lower than that of the conventional PPDI coherent receiver through the excess insertion loss due to these components. The offset was swept from 7.5 GHz to 17.5 GHz at −40 dBm received power, achieving a BER of 10^−3^, as shown in Fig. 4. To minimize the penalty due to the back-reflections in the 3-dB coupler, the optimum spacing between the counter-propagating channels was found to be ≥11 GHz. Significant performance degradation was observed below 11 GHz due to the linear crosstalk between the DS and back-reflected US signals. On the other hand, the coherent receiver and ADC bandwidths were the limiting factors above 14 GHz. Thus, 12.5 GHz, which is compatible with the ITU grid, was chosen as the frequency offset between the DS and US channels. The robustness to linear crosstalk between the DS and US channels was also achieved due to the frequency selectivity of the coherent ONU receiver.

**Fig. 5:**
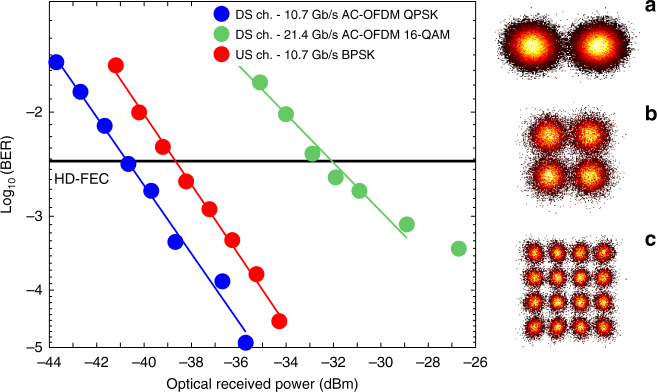
BER vs received power for the DS and US signals in single-channel operation. Insets: received **a** 10.7 Gb/s BPSK, **b** 10.7 Gb/s Alamouti-coded OFDM QPSK, and **c** 21.4 Gb/s Alamouti-coded OFDM 16-QAM constellations around the HD-FEC threshold, assumed to be 4 × 10^−3^. AC-OFDM: Alamouti-coded OFDM

Following the selection of the frequency offset, the receiver sensitivities for the DS (Alamouti-coded OFDM QPSK) and US (BPSK) signals operating at 10.7 Gb/s were measured to be −40.9 and −38.8 dBm, respectively, at the HD-FEC threshold (assumed to be 4 × 10^−3^), as shown in Fig. 5 along with their received constellation diagrams at the HD-FEC threshold shown in the insets (a) and (b). The 2 dB performance difference between the signals was due to the fact that the US transmitter electronics’ bandwidth was limited to 7 GHz using a LPF. The distortion caused by the filtering can also be clearly observed from the eye diagram, shown in the inset (c) of Fig. 3. To further demonstrate the phase-diversity of the proposed polarisation-independent coherent receiver, and increase the achievable spectral-efficiency, the sensitivity performance of Alamouti-coded OFDM 16-QAM with the received constellation at the HD-FEC threshold are also presented in Fig. 5. The required receiver sensitivity at the HD-FEC threshold was found to be −32 dBm. A total sensitivity penalty of 9 dB was observed when the modulation format was switched from QPSK to 16-QAM as it is highly susceptible to laser phase noise and nonlinear distortion caused by clipping compared to QPSK, i.e., 6.7 dB degradation in sensitivity due to the decrease in minimum Euclidean symbol spacing, 0.8 dB due to the limited resolution of DACs/ADC, and 1.5 dB due to the residual phase noise.

### Transmission over 37.6 km

**Fig. 6:**
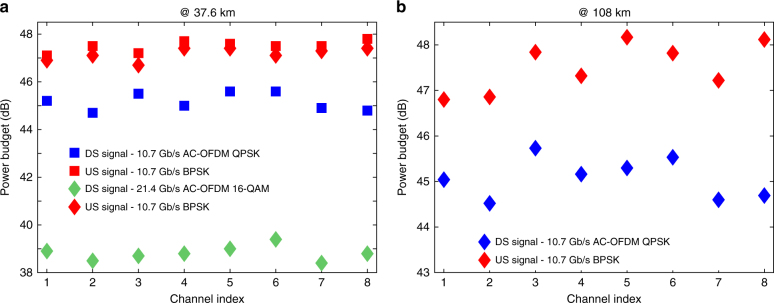
Achieved power budgets for all signals in both transmission scenarios. **a** All DS channels operating at 10.7 and 21.4 Gb/s, and all US channels operating at 10.7 Gb/s over a transmission distance of 37.6 km of installed SSMF. **b** All 10.7 Gb/s DS and US channels over a transmission distance of 108 km of installed SSMF

Initially, the US launch power was optimised while the DS transmitter was switched off to maximise its power budget. The optimum launch power per channel for the US (BPSK) signal was found to be 7 dBm. Once, the launch power for the US signal was determined, the DS transmitter was switched on and its launch power was similarly optimised in the presence of US signal operating at its optimised launch power. The optimum launch power per channel for the DS (Alamouti-coded OFDM QPSK) signal was found to be 4 dBm. A power budget of 44.5 dB (at the HD-FEC threshold) over an installed SSMF at a transmission distance of 37.6 km was achieved for all DS and US channels, as presented in Fig. 6a. Note that the system power budget is determined by the channel exhibiting the worst performance, that is Channel #2. Following the QPSK transmission, the optimum launch power per channel was increased to 6.5 dBm for the Alamouti-coded OFDM 16-QAM signal. Consequently, the achieved power budget was found to be reduced to 38.5 dB due to the higher required received signal power of the Alamouti-coded OFDM 16-QAM signal, but the achieved bit rate for the DS channels was doubled, as shown in Fig. 6a. In both transmission experiments, a variation of ±0.5 dB in achieved power budgets across the channels is observed due to the small power variations between the channels, as can be seen from the optical spectra shown in the insets (a) and (b) of Fig. 3. Assuming that a 3-dB splitter has a typical loss of 3.5 dB, the power budgets of 44.5 and 38.5 dB enable 1:512- and 256-way power splits (the number of subscribers) plus 2.6 and 0.5 dB sensitivity margins over a transmission distance of 37.6 km SSMF, respectively. Such exceptional power budgets were achieved due to the high receiver sensitivity, frequency selectivity, and the robustness to chromatic dispersion that are all facilitated by the coherent technology.

The significance of this demonstration is that it is commensurate with the NG-PON2 standards, which specify a 100 GHz grid and 40 Gb/s aggregate data rate over a transmission distance of 40 km of SSMF. At this transmission distance, our proposed coherent ONU transceiver offers 8 and 4 times higher split ratios (number of subscribers) using Alamouti-coded OFDM QPSK and 16-QAM signals, respectively, compared to the NG-PON2 standards. Thus, such demonstrations provide important markers for comparison. In addition, typically an optical circulator is used to couple the DS and US signals (both in OLT and ONU sides) in research studies even though the integration of a circulator in an ONU transceiver is challenging due to the ONU’s stringent size requirements. Therefore, in practice, it is preferable to use a 3-dB coupler instead of an optical circulator, particularly in the ONU. However, a 3-dB coupler has 20–30 dB less isolation which implies that sensitivity penalties might result from the back-reflected DS or US signals. Due to the high sensitivity and frequency selectivity of the proposed coherent receiver, the isolation provided by the 3-dB couplers was adequate to successfully couple and bidirectionally transmit the DS (both Alamouti-coded OFDM QPSK and 16-QAM) and US signals with negligible sensitivity penalties over a distance of 37.6 km of installed SSMF, as shown in Fig. 6a.

### Transmission over 108 km-long-reach WDM-PON demonstration

The network was next configured to increase the total transmission distance to 108 km (compatible distance for future long-reach PON technology^3, 25^) with 27.6 dB total link attenuation. 10.7 Gb/s Alamouti-coded OFDM QPSK as the DS signal and single-carrier BPSK as the US signal were transmitted bidirectionally at their optimum launch powers (as discussed in the previous section) over a 108 km of installed SSMF. Compared with the previous transmission experiments, similar power budgets for each channel were achieved for this network configuration (Fig. 6b). The scheme offers a 1:16-way power split (number of subscribers) with a 2.4 dB sensitivity margin without the use of mid-span amplification or an opto-electronic extender at a transmission distance of 108 km over the installed SSMF. It is important to note that, although there was no significant linear crosstalk between DS and US channels, the resolution of the sampling scope was dominated by the back-reflected US signal from the 3-dB coupler in the ONU. Therefore, the coupler was replaced with an optical circulator only in the ONU to achieve higher isolation between the DS and US signals. This could be easily avoided by using an electrical band-pass filter (BPF) with a 3-dB bandwidth of 10 GHz having cut-off frequencies of 8 and 17 GHz, which was not available during the experiment. In the OLT, an electrical LPF with a 3-dB bandwidth of 7 GHz was used to filter out the back-reflected DS signal. This demonstration matches well with the typical transmission distance requirements for future generation long-reach WDM-PON systems^25^.

**Fig. 7:**
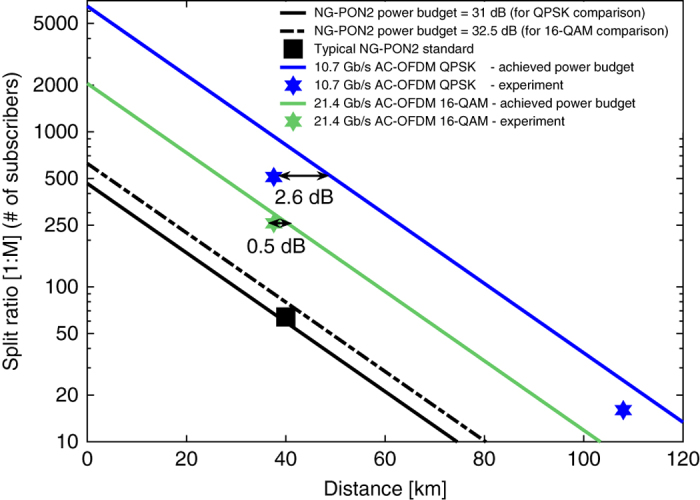
Achievable split ratios with respect to transmission distance. Channel #2, offering the lowest power budgets of 44.5 and 38.5 dB for the Alamouti-coded OFDM QPSK and 16-QAM, respectively, is considered in this analysis. Loss per splitter and average fibre attenuation are assumed to be 3.5 and 0.26 dB/km, respectively. The experimental demonstrations are shown by the *blue* and *green markers*. Note that the margins of 2.6 and 0.5 dB correspond to the sensitivity margins. An extrapolation analysis for the NG-PON2 (with a power budget of 31 and 32.5 dB) is also shown in the plot. The NG-PON2 marker corresponds to 1:64-way power split (allowing 64 subscribers) at a transmission distance of 40 km

We next extrapolated the desired NG-PON2 power budget and the achieved power budgets to distances of up to 120 km to estimate the achievable splitting ratios with respect to the transmission distance, as shown in Fig. 7. In this analysis, the achieved power budgets of 44.5 and 38.5 dB for the Alamouti-coded OFDM QPSK and 16-QAM signals were used whereas the NG-PON2 power budgets were considered to be 31 and 32.5 dB, respectively, due to the difference in launch power per channel in the two demonstrations^2^. The ODN used in this work has 31 dB loss (=3.5-dB per splitter × 64 users + 38 km × 0.26 dB/km), and hence, it can be considered as class N2, allowing a launch power per channel between 5 and 9 dBm, as indicated in Tables 11–5 at p. 24 in ref. ^2^. Note that the loss of 1:2-way power split and the average fibre attenuation were assumed to be 3.5 dB and 0.26 dB/km, respectively. The figure exhibits that the achieved power budgets at a given transmission distance, e.g., say 40 km, can support four and eight times more subscribers than the NG-PON2 standards, respectively. Alternatively, the reach of an optical access network can be extended from 40 km to 65 km and 90 km at a given number of subscribers, e.g., say 64 users, whilst offering 21.4 Gb/s and 10.7 Gb/s per user using QPSK and 16-QAM signals, respectively.

## Discussion

Intensity modulation/direct detection (IM/DD) transceivers using TDM signaling (the currently employed technology) will be ultimately inadequate to offer multi-gigabit/s per user broadband connections, as discussed in the Introduction. In contrast, a WDM-PON architecture offers promising solutions since each user is assigned to a different wavelength, i.e., no bandwidth sharing among the users is required. Moreover, it is scalable to even higher data rates per user and compatible with the existing passive split-based ODN infrastructures.

**Fig. 8:**
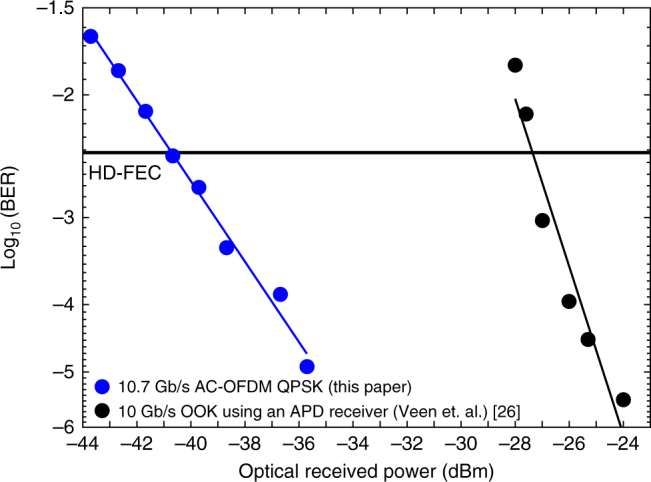
Performance comparison of our PI coherent receiver and a direct detection (APD) receiver. BER vs optical received power for 10.7 Gb/s Alamouti-coded OFDM QPSK received using the PI coherent receiver (demonstrated in this article) and 10 Gb/s TDM-OOK received using a direct-detection (APD) receiver (demonstrated by Veen et al.)^26^

The proposed polarisation-independent (PI) receiver, comprising just a 3-dB coupler and a single balanced photodetector, uses the simplest (minimum) possible optical architecture for a coherent receiver, corresponding to a 75% reduction in the number of photodetectors compared to the conventional polarisation- and phase-diverse coherent receiver whereas the direct detection receiver consists of an optical BPF followed by a single-ended photodiode. The PI coherent receiver is able to operate at a bit rate of 10.7 Gb/s Alamouti-coded OFDM signal requiring just 59 photons-per-bit (−40.9 dBm receiver sensitivity) whereas, to the best of our knowledge, the highest reported receiver sensitivity for a 10 Gb/s TDM-OOK signal achieving the HD-FEC threshold is −27.2 dBm (~1400 photons-per-bit), and require the use of an APD receiver^26^, as shown in Fig. 8.

A direct detection alternative to a coherent receiver, which ostensibly offers a comparable optical complexity, electrical power requirement, and power sensitivity, is a single-photodiode receiver with optical pre-amplification. In this receiver design, the LO laser in the coherent system is substituted for an optical pre-amplifier. For many coherent PON configurations, this would be a reasonable comparison, however for the simplified coherent receiver presented herein, the use of a single laser for both the US signal and LO means that only a single laser is present in the ONU (equivalent to the direct detection ONU without pre-amplification, which requires a laser for US transmission). The simultaneous use of ONU laser comes at the expense of higher electronic complexity (approximately doubling the required ADC bandwidth and sampling rate) compared to a direct detection receiver. The maximum ONU laser power used in the proposed coherent receiver was 14 dBm, which was shared between the US signal and the LO, and is comparable to the maximum laser power used for current PONs. Furthermore, midspan device-based solutions exist to make direct detection solutions more competitive with coherent solutions in terms of maximum reach. For example, arrayed waveguide gratings (AWGs) eliminate mid-span power splitter losses, and enable WDM for direct detection systems. Notwithstanding the complexity of including thermally stabilised AWGs at a remote node, it should be noted that the reach advantages apply equally to direct detection and coherent systems; with the advantage in the coherent scenario that the system is not dispersion-limited and requires no AWGs since coherent detection is inherently wavelength/frequency selective.

**Fig. 9:**
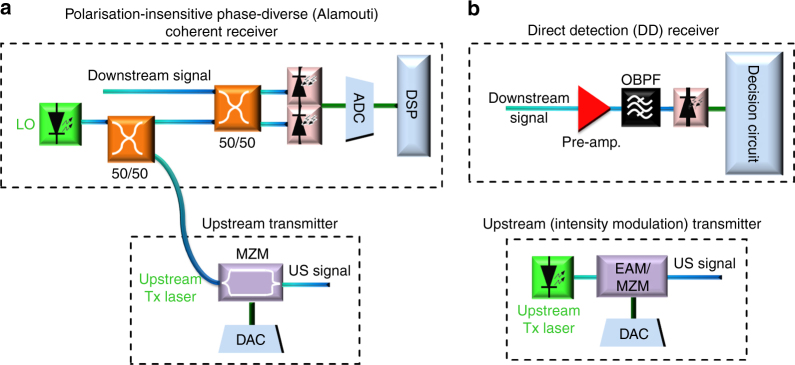
The schematics of simplified coherent and direct detection ONU transceivers. **a** Proposed polarisation-independent coherent ONU transceiver enabled by Alamouti coding combined with heterodyne detection. **b** Typical intensity modulation/direct detection transceiver. Note that analogue processing using a decision circuit is sufficient to demodulate an OOK signal. EAM: electro-absorption modulator, MZM: Mach-Zehnder modulator

The achieved high sensitivity makes the polarisation-independent coherent receiver particularly attractive in such applications. Exceptionally high receiver sensitivity yields an increase in the number of subscribers supported in a network, i.e., reducing the operating cost-per-subscriber. Moreover, the proposed low-complexity coherent ONU is comparable in complexity to the IM/DD ONU transceiver, as shown side-by-side in Fig. 9, as IM/DD ONU transceiver requires a laser to generate an US signal. The other key advantages of the proposed coherent ONU transceiver are: (1) linear optical field detection, yielding high achievable spectral-efficiency (through the realisation of high-order modulation formats, e.g., *M*-QAM) and robustness to chromatic dispersion, enabling longer reach (consolidating back-haul and access spans) in long-reach PONs. Hence, this technology potentially offers further cost savings by extending the physical reach of the access network to the core network using no mid-span amplification or opto-electronic extenders. (2) Frequency selectivity for colourless network operation, i.e., no need for AWGs used as demultiplexers in WDM-PONs. (3) Last but not least, the proposed coherent ONU has the potential to be compatible with the recently approved PON standard NG-PON2, requiring a typical power budget of 31 dB (the proposed solution exceeds this, offering a power budget of 44.5 dB), and it can co-exist with the previous G-PON systems, enabling the gradual migration of subscribers.

The complexity of the proposed coherent ONU transceiver can be further reduced by using a directly modulated laser, removing the need for external modulation such as an electro-absorption or Mach-Zehnder modulator, as demonstrated in ref. ^27^. In addition, if an Alamouti-coded single carrier transmission (rather than OFDM signaling) can be realized for the DS signal, the required DSP for the proposed coherent ONU can be reduced, such as requiring no frame synchronization or FFT operations, yielding lower power consumption. However, the conventional receiver DSP utilised for single carrier systems (2 × 2 multiple-input-multiple-output equaliser followed by carrier phase recovery using Viterbi and Viterbi algorithm^28^) cannot be performed in the case of single carrier Alamouti-coded system due to the conjugation of a symbol in the received symbol-pair, as shown in Eq. (5) in ref. ^22^. Besides this, Alamouti polarisation-time block coding has an orthogonal structure so that it is highly susceptible to phase noise, introducing random rotations, and reduces the block code orthogonality. Hence, either a joint channel and carrier phase estimation scheme needs to be performed, as proposed and tested in simulations in ref. ^29^ or the carrier phase estimation needs to be performed prior to the channel estimation.

## Methods

### Digital signal processing

In the OLT transmitter, the DS signal modulation format was chosen to be Alamouti-coded OFDM QPSK and 16-QAM. The OFDM signal frames were generated offline in Matlab using mutually decorrelated de Bruijn bit sequences of length 2^16^. In both cases, the fast Fourier transform (FFT) size was chosen to be 512, of which 316 subcarriers were found to be sufficient to achieve 10.7 and 21.4 Gb/s per channel, respectively. 18 subcarriers were dropped around the DC frequency (so-called null subcarriers) to insert an optical carrier, which was utilised in frequency offset correction (FOC) and phase noise mitigation. Note that dropping 18 subcarriers was found to be sufficient to mitigate the phase noise, originated from 100 kHz transmitter laser and 1 MHz LO laser, as described in the DSP in the ONU Section below. The carrier was inserted (by biasing the modulator close to its null point) on both polarisation modes to avoid any power fading on the detected polarisation state during fibre transmission due to polarisation rotation for robust FOC and phase noise mitigation. The optical carrier-to-signal power ratio (CSPR) per polarisation was set to −9 dB. Note that the optimum CSPR was found to be the same for both QPSK and 16-QAM signals. Two highly correlated OFDM symbols were inserted on both polarisation modes for the Alamouti-coded OFDM frame synchronization for the same reason, as stated for the FOC and phase noise compensation. Pair-wise training symbols (TSs) (20 of them at the start of the OFDM frame, and 4 of them at every 34th OFDM symbol) were used for channel estimation. In the time domain, Alamouti coding was applied to the orthogonal polarisation states, as discussed in the Principle of operation and Alamouti coding section and detailed in refs. ^20, 22^. Pre-emphasis was applied to optimise the transmitter signal-to-noise ratio, degraded due to the DACs’ finite bandwidth, and subsequently, a 512-point inverse FFT was applied to generate the orthogonal subcarriers. Chromatic dispersion tolerance was achieved using a cyclic prefix, appending 25 samples per OFDM symbol. Finally, the OFDM waveforms were clipped to minimise the distortion due to the DACs’ limited resolution, i.e., setting the peak-to-average-power ratios of QPSK and 16-QAM waveforms to 7.4 and 9.2 dB, respectively. For QPSK and 16-QAM signaling schemes, the raw bit rates of each DS channel were set to 11.3 and 22.6 Gb/s, respectively. In both cases, a 4% overhead cyclic prefix to achieve tolerance to the accumulated chromatic dispersion, and a 5% overhead pair-wise TSs for channel estimation were inserted. This yields total net bit rates of 10.7 and 21.4 Gb/s, respectively, with a HD-FEC threshold of 4 × 10^−3^ being assumed.

The US channels were detected using a PPDI coherent receiver, and digitized using four ADCs (two quadratures for each polarisation) in the OLT receiver. The receiver DSP was performed offline in Matlab. Following de-skewing and orthogonalization, the received signal was resampled to 2 samples-per-symbol. Constant modulus algorithm based adaptive equalisation was applied using a T/2-spaced 11-tap real-valued equaliser, as described in ref. ^30^. The multiple-input-single-output equaliser taps were updated using the least mean squares algorithm. Prior to carrier phase estimation, the intermediate frequency (IF) offset of ~100 MHz was estimated and corrected using the block-based method (removing the data by raising to the second power, and consequently, locating the peak frequency using a FFT over a window of length 1024 samples). Carrier phase estimation was performed by applying the Viterbi-Viterbi algorithm^31^, averaging the complex field over 64T-spaced sliding window to improve the estimation performance. Finally, symbols were mapped to bits making hard decisions, and the BER was estimated by error counting over 2^18^ bits.

### DSP in the ONU

In the ONU receiver, the signal was digitised using a single ADC and processed offline in Matlab. Since the heterodyne detection was used, the received Alamouti-coded OFDM electrical signal was double sideband (real-valued), the frequencies from DC to 7 GHz allocated for the US signal whereas the received DS signal occupied the frequencies from 8 to 17 GHz, and the optical carrier inserted at DC frequency in the DS transmitter appeared as an IF tone. The received signal was down-converted at an IF of 12.5 GHz to re-construct the in-phase and quadrature components. The OFDM frame synchronization was achieved using the Schmidl and Cox algorithm^32^. Following the synchronization, residual frequency offset was corrected by locating the down-converted IF pilot-tone frequency. Since the phase noise distorted the pilot tone exactly the same way as the signal, the pilot tone was filtered using a 5^th^-order Butterworth LPF (a 3-dB bandwidth of 20 MHz), subsequently conjugated and multiplied with the received signal to mitigate the random phase rotations due to the laser phase noise^33, 34^. The cyclic prefix was stripped off and a 512-point FFT was applied prior to channel estimation. Training symbols were utilised to estimate the channel response via the zero-forcing criteria^35^ using an Alamouti decoder^22^, followed by the BER estimation through error counting over 2^18^ bits. There is no DSP required for the US transmitter to preserve the simple implementation of the ONU transceiver.

### Data availability

The data that support the findings of this study are available on request from the corresponding author (M.S.E.). The data are not publicly available due to the research participant privacy/consent.
